# Cohesin biology meets the loop extrusion model

**DOI:** 10.1007/s10577-017-9550-3

**Published:** 2017-02-16

**Authors:** Christopher Barrington, Ronald Finn, Suzana Hadjur

**Affiliations:** Research Department of Cancer Biology, Cancer Institute, University College London, 72 Huntley Street, London, WC1E 6BT UK

**Keywords:** Cohesin, CTCF, Chromosomal domain, Loop extrusion

## Abstract

Extensive research has revealed that cohesin acts as a topological device, trapping chromosomal DNA within a large tripartite ring. In so doing, cohesin contributes to the formation of compact and organized genomes. How exactly the cohesin subunits interact, how it opens, closes, and translocates on chromatin, and how it actually tethers DNA strands together are still being elucidated. A comprehensive understanding of these questions will shed light on how cohesin performs its many functions, including its recently proposed role as a chromatid loop extruder. Here, we discuss this possibility in light of our understanding of the molecular properties of cohesin complexes.

## Introduction

The spatial organization of the genome and the manner in which genes and regulatory elements are embedded therein has an important role in facilitating the regulation of gene expression. The study of the three-dimensional organization of chromatin in nuclear space is transforming our understanding of the mechanisms that regulate gene activity.

Chromosomes are partitioned into spatially demarcated, approximately megabase-sized chromatin interaction domains, termed “topologically associated domains” (TADs) or “chromosome domains” (Dixon et al. 2012a; Nora et al. 2012; Sexton et al. 2012). Organization of chromosomes into domain structures is thought to be important for gene regulation (Noordermeer et al. 2011; Jin et al. 2013; Ghavi-Helm et al. 2014; Giorgetti et al. 2014) and DNA replication (Pope et al. 2014). Domains are maintained across cell types (Dixon et al. 2012a; Nora et al. 2012) and during evolution (Vietri Rudan et al. 2015), further highlighting their functional importance. These discoveries describe a modular organization of chromosomes, embedding thousands of genes in large complex mammalian chromosomes in a structured way. Importantly, it provides a framework within which the effects of distal regulatory elements on gene transcription can be restricted.

Structural maintenance of chromosome (Smc) proteins are major constituents of interphase and mitotic chromosomes and are known to have a key role in mediating chromosome conformation throughout the cell cycle (Nasmyth and Haering 2009). Smc proteins make up both condensin and cohesin complexes. Specifically, cohesin complexes create intra-chromatid contacts for the purposes of sister chromatid cohesion in S-phase as well as inter-chromatid contacts mediating distant-element interactions for the purposes of transcriptional regulation (Hadjur et al. 2009; Mishiro et al. 2009; Wendt et al. 2008) and chromosome domain structure (Sofueva et al. 2013; Zuin et al. 2014a; Seitan et al. 2013) during G_1_ phase. Cohesin complexes facilitate spatial organization by anchoring multiple scales of chromatin loops throughout the genome (Sofueva et al. 2013; Zuin et al. 2014a; Seitan et al. 2013) from high specificity, directional CTCF-bound sequence motifs (Vietri Rudan et al. 2015; Rao et al. 2014).

Recently, a mechanistic model based on in silico simulations has been put forward to explain chromosomal domain formation (Fudenberg et al. 2016; Goloborodko et al. 2016; Alipour and Marko 2012). According to the model, chromosomal domains are formed when “loop extrusion factors” (LEF) translocate along DNA until they encounter a boundary element (BE) that inhibits further translocation. It has been proposed that cohesin proteins may function as loop extrusion factors. Here, we review the current literature with respect to the known molecular properties of cohesin complexes and relate these to its capacity to extrude chromatin loops.

## The cohesin complex

**Fig. 1:**
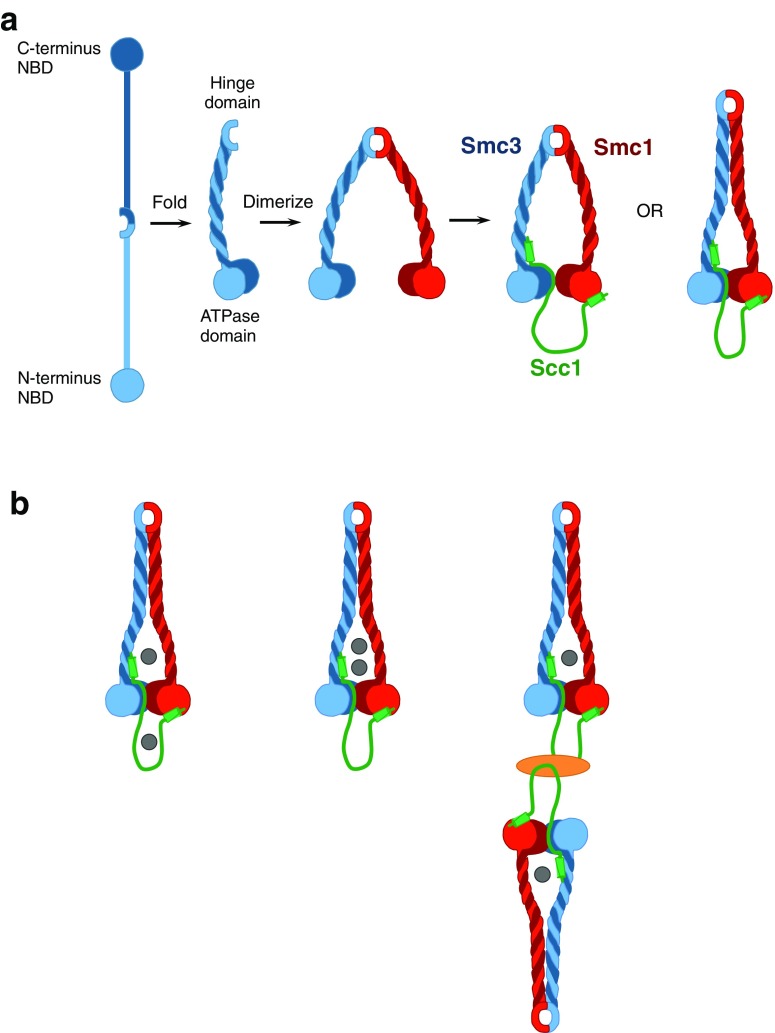
Representations of the structure and conformations of the cohesin ring (not drawn to scale). **a** Smc proteins contain a nucleotide binding domain (NBD) at their N- and C-terminal ends and a central “hinge” domain. The protein folds back on itself to form 50 nm long rod-shaped antiparallel coiled coils bringing the N- and C-terminal NBDs together to form an ATP-binding cassette. Interactions between the Smc hinge domains close the ring on one interface, while asymmetric interactions between Scc1 and the Smcs close the other two interfaces. Evidence exists to support both a fully open and partially open ring structure of cohesin. **b** Based on the loading mechanisms proposed and the different conformations that the cohesin complex may adopt, various possibilities exist for how cohesin can entrap chromatin (indicated by gray circles) to facilitate loop extrusion. *Left panel*, embrace of two strands according to the “two gate” model where one of the strands remains entrapped within the Scc1/Smc pocket. *Middle panel*, embrace of two strands that could be trapped simultaneously when the hinge domain opens and held within the ring. *Right panel*, the handcuff model could also accommodate the entrapment of two strands whereby each strand enters a ring via its hinge domain with interactions between two cohesin complexes mediated by other proteins (*orange*)

The cohesin complex, and in particular its Smc subunits, are deeply evolutionarily conserved proteins owing to their essential and diverse roles in chromosome biology (Hirano 2005). The core complex is composed of Smc1, Smc3, and Scc1 proteins (Michaelis et al. 1997; Gruber et al. 2003). Each Smc subunit has an ABC-like nucleotide binding domain (NBD) at either terminus of the protein and a central “hinge” domain. The protein folds back on itself from the hinge region to form 50 nm long rod-shaped antiparallel coiled coils bringing the N- and C-terminal NBDs together to form an ATP-binding cassette. Heterotypic interactions between the Smc1 and Smc3 hinges create a stable V-shaped Smc1/Smc3 heterodimer (Haering et al. 2002, 2004; Melby et al. 1998). In the presence of ATP, the Smc1 and Smc3 NBDs can engage and then hydrolyze the ATP molecules localized between them (Arumugam et al. 2006) (Fig. 1a). The Scc1/Rad21 subunit of the complex makes asymmetric contacts with the SMCs whereby the C-terminus of Scc1 binds to the Smc1 ATPase domain and the N-terminus binds to the coiled coil region just adjacent to the Smc3 ATPase domain (Haering et al. 2004), and in so doing is thought to form a tripartite ring-like structure (Fig. 1a). Protein cross-linking coupled with mass spectrometry experiments have recently been used to solidify an expansive body of evidence that indeed the Smc1, Smc3, and Scc1 subunits form a closed ring structure (Gligoris et al. 2014; Huis in ‘t Veld et al. 2014). Considering the evidence that cohesin can tether DNA molecules together (Haering et al. 2008), the so-called “embrace” model of cohesin structure posits that cohesin would entrap and thereby topologically anchor DNA strands together within this closed ring (Fig. 1b).

## Cohesin and loop extrusion

Chromosomal domains have been identified across species as diverse as yeast and humans, suggesting that they represent a fundamental organizing principal of chromosomes. While the molecular mechanisms that establish and stabilize these domains remain uncertain, it is clear that chromatin loops are the building blocks of genome structure. Cohesin and CTCF have emerged as key factors in regulating genome structure; CTCF defines a grid of potential interaction sites that, together with cohesin, anchor a global network of chromatin interactions (Sofueva et al. 2013; Zuin et al. 2014a; Seitan et al. 2013).

Recently, an exciting model has been put forward to explain the mechanics of chromosomal domain organization. The *loop extrusion* (LE) model (Alipour and Marko 2012; Goloborodko et al. 2016) succinctly describes domain formation and can recapitulate experimentally derived genome structures (Fudenberg et al. 2016). The simulations upon which the model is based predict that most of the genome will be compacted into consecutive domains, which is corroborated by Hi-C data. According to the model, chromosomal domains are formed when LEFs translocate along DNA until they encounter a BE that inhibits further translocation. An individual BE could be any DNA-bound complex that is sufficiently large or in such a conformation that it physically blocks the LEFs. The authors propose that cohesin (and condensin (Alipour and Marko 2012; Goloborodko et al. 2016; Nasmyth 2001)) proteins may function as LEFs and CTCF proteins as BEs. Here, we discuss the impact of this in silico research on our understanding of the molecular properties of cohesin complexes.

### Cohesin complex topologies

Cohesin’s ring structure appears to satisfy the requirements of a LEF at the heart of the loop extrusion model. Indeed, the structural conservation of SMC-kleisin complexes from bacteria to eukaryotes (Hirano 2005; Wilhelm et al. 2015) raises the possibility that SMC-kleisin complexes could have operated as LEFs throughout evolution, contributing to the formation of the chromosomal domain structures that have been observed in all species studied to date (Nora et al. 2012; Sexton et al. 2012; Crane et al. 2015; Mizuguchi et al. 2014; Dixon et al. 2012b).

According to the loop extrusion model, the LEF must act as a topological device capable of sliding along chromatin. Therefore, it is important to understand the exact nature of cohesin’s ring topology including how wide the opening of the ring actually is. Some of the first electron microscopy observations identified fully open rings, partially open rod-like structures, and oligomers (Melby et al. 1998). Indeed, given all we now know about cohesin’s many roles in the nucleus, it stands to reason that a protein complex with such functional diversity may in fact adopt different conformations influenced by its context-dependent chromatin interactions or post-translational modifications (Skibbens 2016).

Models for cohesin-DNA interactions fall into two main categories. First, the embrace model describes a cohesin ring that is capable of trapping two DNA strands (Haering et al. 2008). The ring can exist with a fully open center (diameter of 35 nm (Huis in ‘t Veld et al. 2014)), or with a partially open center (diameter of 20 nm (Stigler et al. 2016)), such as a rod (Fig. 1a). These conformations may need to accommodate a 30 nm chromatin fiber so subsequent cohesin conformation changes may be necessary to tighten the complex around the fiber. Cohesin could adopt such a rod structure through intra-cohesin coiled coil interactions which have been observed in crosslinking experiments (Huis in ‘t Veld et al. 2014). Further, the coiled coil domains of mammalian SMC proteins are highly conserved (White and Erickson 2009) and harbor mutations linked to Cornelia de Lange Syndrome (Deardorff et al. 2007), supporting their functional importance. Second, the “handcuff” model describes two cohesin rings, where each ring interacts with DNA and each other (Fig. 1b, right panel). Molecular evidence for the handcuff model of cohesin (Zhang and Pati 2015) is supported by studies which show that Scc3 or Pds5 (accessory subunits to the core complex) may act as the factors which structurally stabilize two cohesin rings into a handcuff conformation (Kulemzina et al. 2012; Tong and Skibbens 2015). Importantly, when Pds5 was removed in cells, cohesin levels on chromatin did not change despite changes in sister chromatid cohesion.

**Fig. 2:**
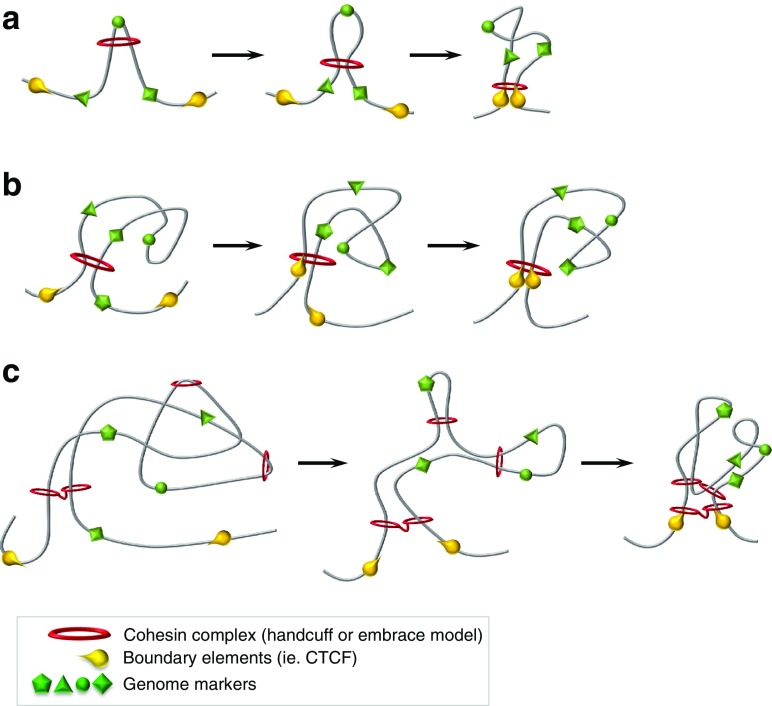
Models for domain formation by cohesin as a loop extruding factor. **a** Cohesin binds within a small genomic region and is translocated in opposite directions up to the BE. The size of domain that would be extruded by this mechanism is likely to be small and could conceivably involve only one loading event. **b** Cohesin binds to two spatially proximal but genomically distant regions of a chromatin fiber. This version could contribute to formation of large domains as observed in Hi-C data. Loading of cohesin in this context would require either rapidly sequential or simultaneous embrace of two spatially proximal strands; loading of two cohesin complexes in spatial proximity which then become associated; or loading of already associated cohesin complexes (as in the handcuff conformation). **c** A combination of the models presented in **a** and **b** could bring about the multiple scales of domain structure observed from Hi-C data

While these models predict the interaction of cohesin and DNA, neither explain the systematic formation of long-range chromosomal contacts to form domains of the scale reported by Hi-C datasets. Interestingly, both the handcuff and embrace conformations of cohesin could satisfy the LE model, and in this context, the interaction of cohesin and DNA may take multiple forms: (1) embrace of a single chromatin fiber and subsequent capture of another, (2) embrace of two chromatin fibers already in near proximity, (3) binding of a pair of associated cohesin complexes in a small region of the chromatin fiber, or (4) binding of associated cohesin complexes to spatially proximal, but genomically distant regions of the chromatin fiber (see Fig. 2 for examples).

### Loading cohesin on chromatin

The LE model does not predict how LEFs might be localized to their loading sites, or even where those loading sites should be relative to the future chromosomal domain to ensure extrusion. Although it is central to our understanding of the LE model and cohesin function in general, a thorough mechanistic view of how the cohesin complex “senses” DNA to then entrap chromatin within its ring remains incomplete.

Loading of the cohesin complex onto chromatin is facilitated by the Scc2-Scc4 complex (NIPBL/Mau2 in humans) (Ciosk et al. 2000) and ATP hydrolysis by cohesin’s ATPase domain (Arumugam et al. 2003; Weitzer et al. 2003; Hu et al. 2011). Scc2 catalyzes the topological loading of cohesin onto DNA in vitro, and loading by Scc2 alone or Scc2-Scc4 did not identify any differences in chromatin association, suggesting that Scc2 may be sufficient for cohesin loading (Murayama and Uhlmann 2014 *)*. On the other hand, crystal structures of Scc2-Scc4 reveal a modular nature for the complex, and deletion mutants show Scc4 is in fact required for efficient Scc2 recruitment to chromatin in vivo. The authors propose that Scc4 may act as a chromatin-adaptor for the Scc2 subunit (Chao et al. 2015). Of note however, low levels of both recombinant human cohesin (Davidson et al. 2016) and yeast cohesin (Murayama and Uhlmann 2014; Çamdere et al. 2016) can be loaded onto DNA in the absence of ATP, albeit inefficiently. Thus, it has been proposed that the loading factors extend the time that cohesin is associated with DNA before it can convert to a topologically bound conformation (Stigler et al. 2016).

If cohesin is loaded in a chromatin-dependent manner, then the chromatin context around loading sites may influence this process. Using ChIP-seq in yeast (Lopez-Serra et al. 2014) and mammals (Kagey et al. 2010; Zuin et al. 2014b), Nipbl binding sites have been found to be enriched at nucleosome-free regions and associated with actively transcribed genes and the epigenetic hallmarks of active genes, including H3K9ac and H3K4me3 and H3K27ac (Lopez-Serra et al. 2014; Zuin et al. 2014b). Furthermore, Nipbl binding sites are enriched for a distinct group of DNA repeats, certain transcription factor motifs, and an oligo(A) motif which is thought to be nucleosome-free (Lopez-Serra et al. 2014). However, insertion of a nucleosome repositioning motif failed to abolish cohesin loading, suggesting that nucleosome positioning alone is not sufficient to inhibit association. Chromatin remodeling complexes were shown to be required for loading onto nucleosome-occupied DNA in yeast (Lopez-Serra et al. 2014).

Gene transcription employs chromatin remodeling so cohesin loading may be linked to active genes through this pathway. Indeed, Stigler et al. provide direct evidence to support transcription-induced mobility for the localization of cohesin at convergent genes (Stigler et al. 2016). The Mediator complex, which is part of the transcription machinery and formed at active genes, was shown to be correlated with Nipbl and Smc1a binding sites using ChIP-seq and later by co-purification in mouse embryonic stem cells, linking transcription with cohesin loading (Kagey et al. 2010). By comparison, ChIP-seq data for a different NIPBL epitope indicates that NIPBL binding sites had very little overlap to cohesin or CTCF sites in human cell lines (Zuin et al. 2014b), which may be explained by the translocation of cohesin to distal loci relative to its loading sites (Lengronne et al. 2004). Whether the differences between the NIPBL ChIP-seq observations is due to technical differences, such as the antibody and experimental protocol, is unclear.

Taken together, these studies provide evidence that the interaction between Nipbl and cohesin facilitates its loading onto DNA and that Nipbl may require a specific set of genomic conditions to bind. Furthermore, Zuin et al. observed that during the cell cycle, NIPBL as well as CTCF bind to the genome before cohesin. In the context of the LE model, this may be interpreted as a network of initiation and termination nodes being defined before genome structure is re-formed following compaction for cell division. The position of the cohesin loading sites and the distances that must be translocated to a boundary element influence the genomic structure. For example, a cluster of loading sites or sparse boundary elements may lead to a large domain formed with many highly compacted sub-domains, whereas a single loading site flanked by boundary elements could lead to a reinforced large-scale domain.

The LE model does not advance our knowledge of the cohesin loading process; however, it does not negate any of the proposed evidence-supported mechanisms. Furthermore, current data does not preclude the existence of multiple concurrent cohesin loading mechanisms. It may be possible that the initial formation of large (approximately megabase-scale domains) is initiated by one mechanism that can form the scaffold around which more specific and definitive domains form across shorter scales, potentially influencing cellular processes and lineage.

### Trapping chromatin within a ring

When cohesin is not engaged in cohesion and thus stably associated with chromatin, it is cycling between DNA entrapment and release (Gerlich et al. 2006). As discussed above, DNA entrapment is dependent on the loading complex Scc2/Scc4, while release depends on the cohesin regulator Wapl (Kueng et al. 2006). As cohesin is a ring structure, loading and release involves opening of the ring by dissociation of one of its interfaces. Two models have been proposed for how cohesin facilitates entrapment of DNA into the complex with distinct entry gates for cohesin. The first predicts that DNA enters and exits through the same gate, whereas another predicts that there are different gates for entry and exit from the complex.

In fission yeast, elegant in vitro reconstitution experiments of cohesin onto plasmid DNA have proposed that DNA enters and exits the cohesin ring through the ATPase head domains of Smc1-Smc3 (Murayama and Uhlmann 2015). Both the entry and exit of DNA into the cohesin ring was demonstrated to take place sequentially, whereby the DNA passes through two “interlocking gates” in defined stages. For DNA to exit, “DNA-sensor” lysines on the inside of the Smc3 ATPase head domain trigger ATP hydrolysis. This interrupts the interactions between the Smc1-Smc3 heads, forcing them apart and allowing the DNA to exit the Smc1-Smc3 ring into a cavity between Smc1-Smc3 and Scc1. Once inside this pocket, Wapl facilitates the disassociation of the Scc1 N-terminus (Scc1N) from Smc3, allowing for the complete exit of DNA from the cohesin ring. Wapl only mediates this Scc1N disassociation when ATP is bound by the Smc subunits (Murayama and Uhlmann 2015).

According to this model, entry of DNA into the ring is believed to be the reverse of the exit reaction with one major difference—the DNA must come into contact with the DNA-sensing lysines from the outside. To overcome the traditional planar ring structure of cohesin, the authors propose that the DNA-sensor lysines are exposed to the outside when the Smc1-Smc3 hinge and Smc1-Smc3 head domains are brought into close contact by the cohesin loaders, Scc2-Scc4. The contact between the hinge domains and the head domains induce an “inside-out” cohesin ring conformational change that exposes the DNA-sensing lysines. A conformational change is supported in the literature by AFM observations and a discernable in vivo FRET signal between fluorophores located on the Smc1-Smc3 hinge and Pds5 located next to the Smc3 ATPase head domain (Mc Intyre et al. 2007).

An alternative model proposes that cohesin has separate DNA entry and exit gates (Nasmyth 2011) whereby the tripartite ring traps DNA via an entry gate at the Smc1–Smc3 hinge interface and releases DNA via an exit gate at the Smc3–Scc1 interface. This model is supported by evidence that by artificially holding the hinge domains together but not by preventing Scc1’s dissociation from SMC ATPase heads, cohesin’s association with chromosomes is blocked (Gruber et al. 2006). Irreversibility of the entry gate is proposed to involve a mechanism by which the presence of DNA within the ring regulates opening, and acetylation acts to prevent further loading events (Nasmyth 2011). The exit gate is proposed to be distinct from the entry gate and located at the interface connecting the ATPase domain of Smc3 with the N-terminus of Scc1 (Eichinger et al. 2013; Buheitel and Stemmann 2013). Recently, cohesin’s release from DNA was found to involve a highly conserved asymmetric activity associated with one its ATPase sites (Elbatsh et al. 2016).

Both of the models for loading cohesin onto DNA use the embrace model as their conformation of choice. However, given the possibility of multiple conformations of cohesin rings, how could the possible entrapment mechanisms influence domain formation by loop extrusion? First, two strands which are genomically and spatially proximal could be trapped when the hinge domain opens and held within the ring embrace. The simultaneous entrapment of two DNA strands would prevent the loss of the first strand when the hinge opens to capture the second if chromatin were loaded sequentially. The embrace of two strands could also occur in the “two gate” model; however, this would require two rounds of ATP hydrolysis and conformational change to occur, presumably while the first trapped strand was held within the ring. Alternatively, the handcuff model could also accommodate the entrapment of two strands which are genomically distal but spatially proximal and fulfill the requirement for loop extrusion. In this version, loading of DNA through the hinge domains of the associated cohesin rings would successfully entrap single DNA strands and extrude chromatin without the need to induce conformational changes of the two rings simultaneously.

### Sliding

An important element of the LE model posits active translocation of cohesin rather than passive diffusion or static interaction. To date, there is no evidence to support the active movement of cohesin, nor for cohesin subunits to possess motor capacity capable of independently facilitating chromatin loop formation. The cohesin complex does indeed have ATPase activity; however, it is weak (Arumugam et al. 2006), even when cohesin is associated with Scc2-Scc4 (Murayama and Uhlmann 2014). To date, cohesin’s ATPase activity is known to regulate structural rearrangements in cohesin itself; the ATPase is required for the dimerization of the Smc1 and Smc3 head domains and to promote loading and release from DNA, possibly by driving conformational changes in the structure (Arumugam et al. 2006, 2003; Weitzer et al. 2003; Hu et al. 2011; Murayama and Uhlmann 2014; Çamdere et al. 2016; Elbatsh et al. 2016; Ladurner et al. 2014). In addition to requiring ATPase activity to associate with DNA, cohesin also exhibits ATPase activity after it is stably bound to DNA which may be important to promote cohesion (Çamdere et al. 2016).

While no evidence exists to suggest cohesin can actively move on chromatin, strong evidence has emerged that once topologically loaded onto chromatin, cohesin can indeed slide along a DNA template in vitro by passive diffusion (Stigler et al. 2016; Davidson et al. 2016). Sliding stops when Scc1 is cleaved, confirming that cohesin is topologically bound to DNA, or when it encounters a protein barrier such as CTCF (Davidson et al. 2016). Importantly, distinctly labeled complexes were not observed to switch positions while sliding along DNA in vitro, indicating that two cohesin complexes cannot bypass one another on DNA (Davidson et al. 2016). Diffusion of cohesin was greatly restricted by higher density nucleosome arrays, and predictions of its movement along crowded physiological settings calculate a 3000 X reduction in processitivity relative to its movement on naked DNA (Stigler et al. 2016).

A model system that relied on passive diffusion of cohesin along DNA estimates that within an hour, cohesin could translocate 7 kb (Stigler et al. 2016). Computational analysis suggests that cohesin needs to translocate up to 500 kb to form a chromosomal domain, leading to an estimated rate of 50 kb/min. The question remains then of what protein(s) could provide the motor force required for translocation and precisely how such a motor interacts with cohesin, including whether cohesin is pushed or pulled. To reproduce the large domains of hundreds of kilobases observed from Hi-C data, loading of cohesin onto DNA at positions close to boundary elements may alleviate the low processivity and together with active translocation permit large domains to form.

### Boundary elements

In the LE model, BEs could constitute a wide range of DNA-bound protein complexes, including known domain proteins such as CTCF, large protein complexes such as Mediator as well as cohesin complexes which are translocating convergently. Importantly, the size of a BE that is sufficient to block cohesin is dependent upon the size of the diameter of the pore, which is itself dependent on the conformation that the complex itself takes (ie. rod vs ring). Using tethered DNA to monitor cohesin translocation along DNA, Stigler et al. determined that the functional pore size of cohesin in its DNA-bound conformation is larger than 10.6 nm but less than 19.5 nm and that FtsK (a 13 nm motor protein) could push cohesin along DNA. These observations led the authors to propose that cohesin could be adopting conformations which alter the confomation of the ring into a rod-shaped structure and thus influence its ability to translocate. Equally, translocation could be inhibited if DNA was entrapped in the space between the Scc1 and the Smc head domains, as proposed by Muruyama et al. Stigler and colleagues addressed the important question of the effect of nucleosomes on translocation and showed that free diffusion of cohesin on chromatin is highly restrictive. Thus, local genomic context, such as sequence composition and epigenetic modification, may impact domain formation and definition in an in vivo context by regulating cohesin translocation.

A prominent feature of cohesin/CTCF-mediated chromosome structure is the specificity of long-range interaction partners (Sofueva et al. 2013). These observations led to the discovery that the selectivity of interaction partners is explained by the orientation of the CTCF sequence motifs at the interacting sites, whereby chromatin loops predominantly form between sites with convergent CTCF motifs (Vietri Rudan et al. 2015; Rao et al. 2014). Why cohesin is most often blocked by convergent CTCF binding sites is not known. The structural conformation of CTCF on DNA is conferred by the orientation of the CTCF motif (Nakahashi et al. 2013). If so, it would be possible that CTCF and cohesin can only interact when cohesin translocates towards CTCF from a specific direction, such as from within the forming domain. Surprisingly, little is known about the molecular interactions between CTCF and cohesin. However, it is understood that the interaction is mediated between the C-terminus of CTCF and the Stag subunit of cohesin (Xiao et al. 2011). Given the importance of these factors to chromosome topology, it will be important to understand the exact nature of their interactions.

### Putting it all together—domain formation by cohesin as a LEF

Considering the LE model and models for cohesin loading, the size of a formed domain would be dependent on the number and position of loading events and boundary elements, rate of cohesin processivity, and the propensity of cohesin to dissociate.

One possibility is that cohesin binds within a small genomic region, in an embrace or handcuff, and is translocated in opposite directions up to the boundaries (Fig. 2a). If the motor force is exerted by an extrinsic factor pushing cohesin, that factor would need to bind DNA at the cohesin complex. The physical space available for motor protein binding is limited by the separation of cohesin rings in the handcuff variant and the size of the nascent loop in the embrace variant. Either way, the physical space available would be small, raising the question of whether large complexes (i.e. RNA polymerases) would fit within this space. The size of domain that would be extruded by this mechanism may be smaller, since the initial genomic separation is very small and the conditions permitting translocation may be rate limiting.

Equally, in an embrace or handcuff model, two spatially proximal but genomically distant regions of a chromatin fiber could become bound (Fig. 2b). Once motor force is exerted on the two cohesin complexes, a loop may be formed that, according to the LE model, can be reinforced, further subdivided, or ultimately lost. If the initial interaction is between vastly distant regions, the interaction is very unlikely to be reinforced by additional cohesin because loading within the large domain would prevent its further reinforcement. Similarly, initial interactions between chromosomes could occur by random chance but would be considered transient, as they are unlikely to form an interaction that can persist. In the context of the handcuff conformation, the two cohesins would need to be associated either at the point of loading or very closely thereafter in order to ensure extrusion. Whether NIPBL can load multiple cohesin rings or contribute to their association once loaded is unknown. However, such a mechanism would alleviate the physical space constraints on motor protein binding, feasibly permitting any processive protein complex to translocate cohesin, and additionally contribute to formation of large domains as observed in Hi-C data.

A combination of the two loading mechanisms could lead to the formation of nascent long-range domains that become sub-divided into smaller sub-domains. By interacting with two genomic regions that are spatially proximal but genomically distal, a domain of considerable size may be created. Loop extrusion can then further increase the size of the domain, limited by BEs, to domains of hundreds of kilobases as observed in Hi-C data. Continuous cohesin loading and loop extrusion could continue witihin the forming domain and be terminated by the same BE as the larger domain on one face and the translocating cohesin on the other (Fig. 2c). This model could prevent the reinforcement of the initial long-range domain since newly loaded cohesin would reinforce the smaller domains. However, Hi-C data shows long-range contacts between distal ends of sub-domains. Given the patchwork nature of neighboring domains predicted by the LE model, the BEs that formed the initial long-range domain may be kept in spatial proximity by its neighboring domains, and thereby be identified during the ligation stages of the Hi-C protocol.

## Future perspectives

While it is tempting to consider cohesin complexes as loop extruding proteins, it is clear that many outstanding questions still need to be addressed. The precise molecular mechanisms leading to DNA entrapment and how this process is regulated are major unanswered questions in the field. Similarly, stability of cohesin on chromatin will clearly impact our understanding of how loops can be extruded, and studies have only begun to shed light on this level of regulation. In this review, we have not discussed the myriad accessory proteins and post-translational modifications which regulate cohesin’s functions; however, understanding how these work together to influence cohesin’s (dis)association with chromatin will be required in order to have a complete picture of loop extrusion by cohesin. Finally, if indeed cohesin and CTCF act as the key factors in a model for domain formation by loop extrusion, then the field urgently requires deeper molecular insights into their interaction on chromatin.

## Abbreviations

Smc: Structural maintenance of chromosome
NBD: Nucleotide binding domain
TAD: Topologically associated domains
LE: Loop extrusion
LEF: Loop extrusion factor
BE: Boundary element
